# Reduced-credit bachelor’s degrees and the BSPH: what should undergraduate public health optimize for?

**DOI:** 10.3389/fpubh.2026.1847063

**Published:** 2026-06-03

**Authors:** Kahler W. Stone, Bethany A. Wrye

**Affiliations:** Department of Health and Human Performance, Middle Tennessee State University, Murfreesboro, TN, United States

**Keywords:** 90-credit bachelor’s degree, abridged bachelor’s degree, bachelor of science in public health, BSPH, CEPH accreditation, public health workforce development, reduced-credit degree, undergraduate public health education

## Abstract

Undergraduate public health education has expanded rapidly in the United States and now serves several overlapping purposes. The Bachelor of Science in Public Health (BSPH) may function as an entry point into the public health workforce, a foundation for graduate public health study, a pathway for students considering clinical professions, and a broad liberal education experience centered on population health. Recent accreditation changes within the Southern Association of Colleges and Schools Commission on Colleges (SACSCOC) have opened a formal pathway for reduced-credit bachelor’s degrees, typically requiring 90–100 semester credit hours, creating a timely opportunity to reconsider what undergraduate public health degrees are intended to do. Drawing on published curriculum analyses of CEPH-accredited programs, employer expectation data, and our own experience in a regional public university BSPH, we argue that the BSPH presents a particularly difficult case for reduced-credit design: its value depends not on any single function but on a curricular structure broad enough to accommodate students whose professional goals evolve across the undergraduate years. We offer a typology of three BSPH model types—workforce-entry, pre-professional/pre-graduate, and liberal arts/population health hybrid—grounded in CEPH 2024 Criterion B1 required curriculum domains, and use this framework to show that compression decisions are unavoidably decisions about which students and functions the degree will prioritize. Shorter structures may suit students who arrive with settled professional goals, but carry real risks: reduced scientific depth, a thinner general education foundation, and a poor fit for transfer, exploratory, and late-deciding students who constitute a meaningful share of most programs.

## Introduction

Undergraduate public health education is no longer a peripheral enterprise. Over the last two decades, the field has matured from a small movement into an established component of public health education in the United States, with a growing number of accredited baccalaureate programs and a broader recognition that public health knowledge, skills, and ways of thinking belong at the undergraduate level (1–3). This growth has been accompanied by a persistent conceptual question: what, exactly, is the undergraduate public health degree for? (2, 4–7).

That question has become more urgent as institutions face pressure to reduce costs, shorten time-to-degree, and offer more visibly workforce-aligned credentials. In March 2026, SACSCOC publicly clarified its pathway for reduced-credit undergraduate degrees, allowing bachelor-level programs between 90 and 100 semester credit hours under specific conditions (8, 9). The framework is not intended to replace the traditional 120-credit bachelor’s degree. Rather, it invites institutions to consider whether some degrees can achieve their objectives through a more focused curriculum while still preserving appropriate rigor, proportional general education breadth, and public transparency.

This policy conversation is not confined to the Southern region. Nationally, multiple institutions and accrediting bodies have begun exploring or approving reduced-credit bachelor’s degree models, particularly where programs are structured around demonstrated learning outcomes and workforce-relevant competencies rather than elective accumulation alone. For example, the Northwest Commission on Colleges and Universities approved several 90–94 credit bachelor’s degree programs at Brigham Young University–Idaho and Ensign College (10), reflecting a growing willingness among some accreditors to evaluate degree sufficiency through evidence of student learning rather than adherence to a universal 120-credit norm alone.

For undergraduate public health, that invitation is both intriguing and destabilizing. The BSPH has historically carried more than one function. It may prepare students for direct entry into public health practice, support later graduate training, provide a nontraditional pre-health pathway, or serve as an applied liberal education major built around population health, systems thinking, and social context (2, 3, 5–7, 11). In our experience within a CEPH-accredited BSPH program at a regional public university, these functions are not abstract categories. They are embedded in advising conversations, curriculum design, recruitment, and students’ evolving academic plans. That context is especially relevant in regional public university settings, where affordability, transfer mobility, and evolving student goals often intersect more visibly with curricular design decisions. A reduced-credit BSPH may therefore appear efficient on paper while forcing difficult decisions about what the degree is fundamentally supposed to optimize.

**Figure 1:**
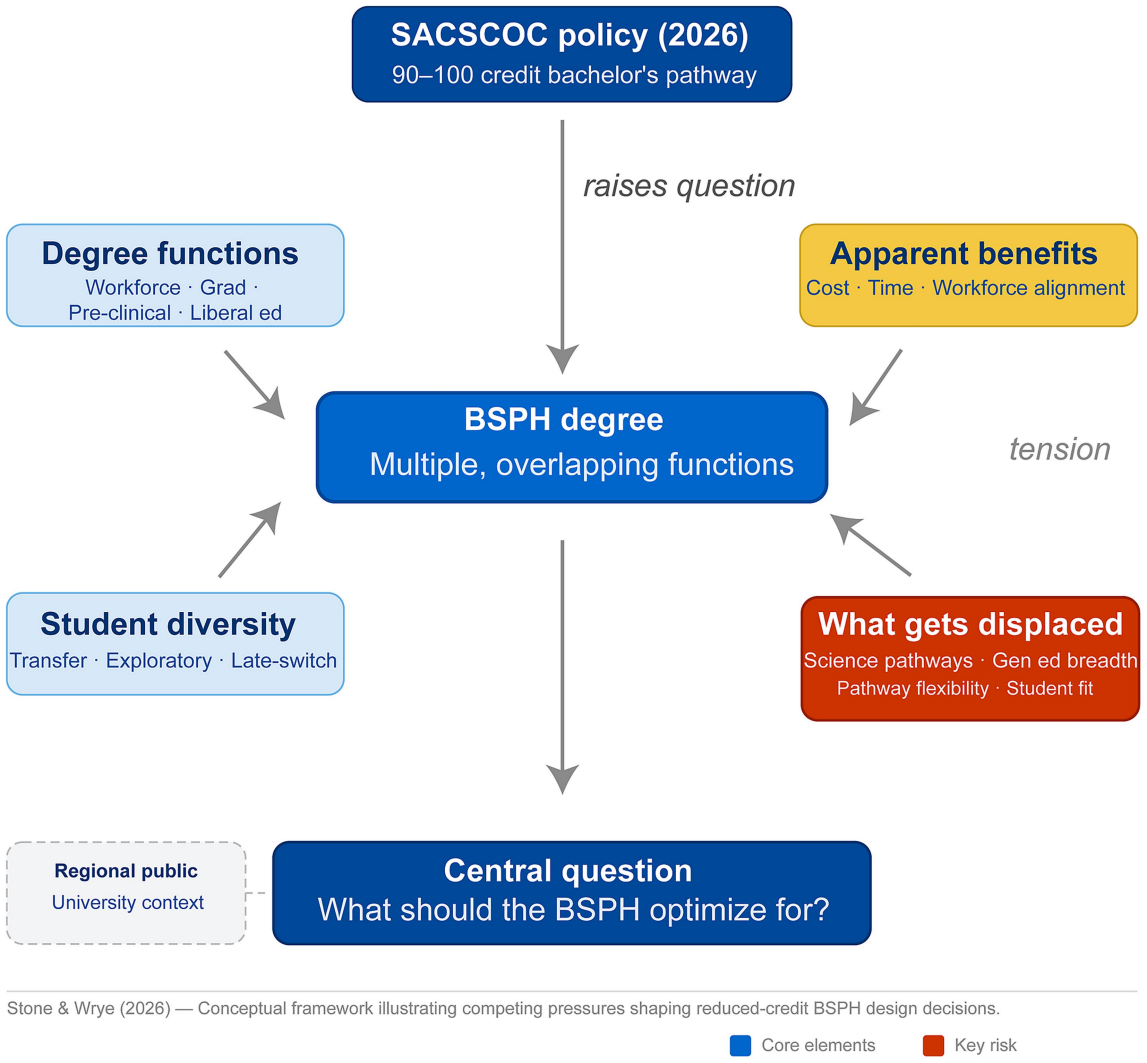
A conceptual framework illustrating competing pressures shaping reduced-credit BSPH design decisions.

Calls for innovation in public health teaching and learning are not new (12); what the reduced-credit pathway introduces is a concrete institutional mechanism that requires programs to act on those calls explicitly, and with documented intent. In this perspective, we argue that reduced-credit bachelor’s degree models may offer appealing efficiencies, but in undergraduate public health, they also raise underexamined questions about workforce signaling, student mobility, disciplinary breadth, and what the BSPH is fundamentally designed to do. We do not argue that a reduced-credit BSPH is inherently inappropriate. Rather, we argue that the BSPH is a particularly poor candidate for *casual* compression (Figure 1). This perspective offers a practice-informed policy critique, arguing that compression decisions for the BSPH are fundamentally decisions about purpose—and that the field must be willing to name that purpose explicitly before structural reforms proceed.

Figure 1 illustrates the structural problem this paper addresses: reduced-credit BSPH design decisions do not occur in a vacuum but at the intersection of multiple competing pressures that pull in structurally incompatible directions. The outer ring of pressures — institutional efficiency, workforce demand signaling, graduate pipeline expectations, and the public health accreditation framework — converge on a degree that has historically absorbed all of them simultaneously. The figure’s decision pathways make visible what compression requires programs to do implicitly: choose which pressure to prioritize, and accept the tradeoffs that follow. A program that optimizes for the top-left pathway (direct workforce efficiency) gains credential specificity but loses the flexibility that serves transfer and late-deciding students. A program that preserves the right-side pathway (broad scientific and graduate pipeline preparation) keeps its pre-professional value but sacrifices the efficiency gains that motivated compression in the first place. The framework is not prescriptive. It does not tell programs which pathway to follow. It does insist, however, that the choice is real — and that the field should make it explicitly rather than absorbing it quietly into a shortened credit count.

## The BSPH is asked to do many things at once

The contemporary undergraduate public health curriculum is broad by design. CEPH’s standalone baccalaureate criteria require exposure to foundational public health learning across statistics, biological and life sciences, the science of human health and disease, determinants of health, project implementation, health systems, law and ethics, and public health communication, alongside information literacy, communication competency, and experiential learning (13). This framework does not define a narrow technical credential. It describes an interdisciplinary degree intended to produce graduates who can think across systems, evidence, populations, and practice. To be precise about what compression threatens: CEPH’s 2024 Criterion B1 specifies 15 distinct required curriculum domains. These span the discipline’s historical and social justice foundations (B1.1–2); the determinants and ethics of health (B1.3–4); biological science and disease foundations that support pre-professional and graduate pathways (B1.5–6); health systems and global health contexts (B1.7–8); quantitative methods, statistical literacy, and surveillance (B1.9–10); applied intervention planning and community partnership (B1.11–12); critical thinking in practice (B1.13); and legislative processes and policy analysis (B1.14–15). Criterion B2 adds six required foundational competencies — each assessed individually — spanning the full arc of public health information use from locating and evaluating evidence to synthesizing it and communicating it to non-specialist audiences. Criterion B4 adds seven workforce preparation concepts including cultural humility, leadership, ethical self-reflection on practice, and flexibility. Criterion B5 requires experiential learning. These are not decorative additions. Each represents a deliberate investment of credit-bearing space that, in a compressed degree, must be justified against every other commitment in an already contested curriculum.

Recent empirical work puts this breadth in concrete terms. In a narrative review of 90 CEPH-accredited undergraduate public health programs, Kedia et al. (14) catalogued 2,259 unduplicated public health courses across 86 institutions, finding that programs most commonly emphasized foundations of public health, epidemiology, management and leadership, environmental health, global health, and social determinants. When mapped onto ASPPH and CEPH domains, two-thirds of course themes aligned with foundational and theoretical concepts, while one-third fell to more practical applications. The “overview of public health” domain alone accounted for 22.5% of course themes, followed by determinants of health (14.6%), human health and disease (13.0%), and health policy, law, and ethics (12.6%). By contrast, data science (6.7%), project implementation (6.0%), and health systems (3.4%) were among the least represented. That split is telling. The same research suggests that programs may need to expand their practical offerings to better meet workforce expectations, not contract them. A degree already navigating that tension has little room to spare. Compressing to 90 credits while simultaneously trying to become more practice-relevant is less a reform than a contradiction.

That architecture matters because students use the BSPH in different ways. Some enter undergraduate public health intending to work in health departments, nonprofit organizations, community health settings, or prevention programs. Others are attracted by the degree’s relevance to medicine, physician assistant studies, pharmacy, dentistry, nursing, or other health professions. Still others discover public health after entering college through a general education course, an introductory health course, a change in major, or a desire for a health-related pathway that is not narrowly clinical. In other words, the BSPH often functions as a degree of layered possibility rather than a single-purpose credential.

This multiplicity has long been both a strength and a source of ambiguity. Kiviniemi and Mackenzie argued that undergraduate public health is best understood, at least in part, through a liberal education frame, one that emphasizes critical thinking, problem solving, and a broad conception of public health citizenship rather than narrow occupational preparation alone (6). That framing remains persuasive. Yet it coexists uneasily with workforce rhetoric, recruitment messaging, and increasing institutional pressure to demonstrate rapid return on investment. Reduced-credit degree proposals intensify this tension because they demand clearer prioritization.

## What reduced-credit BSPH models appear to promise

The appeal of reduced-credit bachelor’s degrees is not difficult to understand. At least in principle, they may lower total tuition burden, reduce time-to-completion, and improve the perceived efficiency of degree attainment (8, 9). For some students, especially those entering college with a clear occupational endpoint and limited interest in broad academic exploration, a shorter pathway may be attractive. Public health programs also operate in a competitive enrollment environment. A reduced-credit model might seem like a mechanism to distinguish the degree, improve affordability, and align with institutional concerns about completion and student debt.

From a curricular standpoint, the reduced-credit conversation can also be healthy. It pushes programs to distinguish essential learning from inherited structure. Many undergraduate degrees contain historical accumulation, legacy course expectations, or distributional habits that persist more by tradition than by evidence. Asking what is truly indispensable can be productive. In public health, it may also encourage more deliberate scaffolding of competencies, fewer redundant requirements, and stronger alignment between course sequences and intended student outcomes.

There is also a plausible workforce argument. SACSCOC’s guidance specifically envisions reduced-credit degrees in specialized or workforce-related fields that prepare graduates for direct entry into employment (8, 9). Some public health roles do not require a heavy advanced science sequence and may benefit more from applied communication, data interpretation, community engagement, program planning, and policy literacy than from additional coursework in chemistry or physics. For a subset of students seeking near-term employment in health promotion, prevention, outreach, or community-based public health settings, a shorter and more focused bachelor’s pathway could be rational.

These arguments should not be dismissed. But they also should not be treated as self-evidently transferable to the BSPH without closer scrutiny.

## What may be displaced when the BSPH is compressed

The first challenge is that public health workforce readiness is not synonymous with curricular reduction. Contemporary practice expectations are often broader, not narrower, than programs assume. Education for Public Health 2030 called for transformation in public health education around leadership, systems thinking, communication, data capacities, and flexibility in a changing environment (7). Similarly, a 2025 Tennessee Department of Health academic-practice symposium involving public health faculty, agency leaders, and accrediting representatives identified student readiness domains that extended well beyond disciplinary content alone, including applied research, critical thinking, data management, policy and advocacy, professionalism, financial management, career development, public communication, and ethical use of artificial intelligence (15). While this report does not represent national consensus, it illustrates a practical reality that is highly relevant to our institutional setting: workforce readiness in public health increasingly depends on integrative, cross-cutting abilities that are often developed across a full undergraduate experience.

The second challenge is pathway flexibility. A recent pilot study by Brown et al. (11) documented substantial variation in required basic sciences across southern BSPH and public health-related programs and argued for more intentional science pathways for students interested in clinical professions. Of the 38 accredited southern programs reviewed, only 12 (31.6%) required introductory biology and 9 (23.7%) required anatomy and physiology; organic chemistry and genetics were each required by a single program, and physics by only two. That is not random variation. Their study is useful because it clarifies two realities at once. First, advanced sciences such as organic chemistry, physics, and genetics are not uniformly required in BSPH curricula. Second, those courses remain consequential for students whose goals extend beyond direct public health employment. The issue is not uniformity. Not every BSPH student needs the same science sequence. The issue is that a shorter degree leaves no room to accommodate that variation without pushing students toward earlier, and potentially premature, decisions.

The third challenge concerns general education. In efficiency-oriented conversations, general education is often treated as the most obvious place to cut. Yet for public health, many general education disciplines are not peripheral. Writing, statistics, sociology, psychology, political science, economics, history, and communication all contribute directly to how students learn to understand behavior, institutions, inequity, evidence, governance, and public trust. SACSCOC’s framework explicitly requires proportional breadth in general education outcomes, including critical thinking, communication, and problem solving, even within reduced-credit models (8, 9). That requirement is not merely procedural. It reflects an educational truth that is especially salient in public health: the field’s practice demands often depend on the very forms of reasoning that broad undergraduate study is supposed to cultivate.

The fourth challenge is student fit. In our setting, the BSPH is not used only by students who enter college with a fixed four-year plan. It is also used by transfer students, by students who discover public health after beginning in another major, and by students whose goals evolve through exposure to coursework, practice experiences, or advising. A reduced-credit model may function best for direct-entry students with a clear endpoint, but it may be less helpful for exploratory students and for those who need time to learn what public health is and how it connects to their interests. That is not a minor concern. Public health often attracts students precisely because it allows them to connect health, service, science, and social context without requiring immediate specialization.

A related but distinct concern is standalone credential viability. Not all reduced-credit undergraduate models are designed primarily as workforce-terminal bachelor’s degrees. Some are structured instead as accelerated 3 + 1 or embedded graduate pathways, in which the undergraduate credential functions partly as a transitional stage toward an MPH or other graduate degree. That structure may work well for students who progress as intended, but it also raises an important student-centered question: what happens when they do not? In public health, where the bachelor’s degree often serves as a meaningful workforce credential in its own right, programs should be cautious about adopting reduced-credit structures that depend too heavily on successful graduate progression. Students who are not admitted, change direction, or experience financial or personal interruption should still have a clear and usable pathway to a complete and independently valuable undergraduate degree.

## Lessons from one regional public university context

These tradeoffs become more visible when viewed through the realities of a regional public university BSPH program, where curricular design is shaped not only by accreditation and workforce expectations, but also by affordability pressures, transfer pathways, and students with divergent and evolving professional goals. Like many regional public university programs, our BSPH serves a mixed student population that includes direct-entry majors, transfer students, students shifting from other health-related pathways, and students using the degree for both workforce and pre-professional aims. In our institutional context, the BSPH is asked to balance affordability, accreditation, workforce responsiveness, transfer realities, and student pathway flexibility at the same time. Students arrive with diverse intentions. Some seek direct entry into public health work. Some view the degree as preparation for later MPH or DrPH study. Some are drawn to the degree because it can complement clinical aspirations while also offering a broader population health perspective. Others find public health later in college after beginning elsewhere. Advising, therefore, is not simply about moving students through a fixed sequence. It is about helping them navigate a degree that must remain usable across several plausible futures.

Within that context, compression is not neutral. Removing credits would not simply eliminate excess. It would require a decision about which student uses of the BSPH deserve priority. If a reduced-credit BSPH were optimized primarily for direct workforce entry, it might reasonably emphasize practical communication, program planning, data use, professionalism, and applied experiences. But that choice could narrow room for additional sciences, reduce elective flexibility, and make the degree less adaptable for students who later pursue clinical or graduate pathways. If, alternatively, the degree preserved wider scientific and pathway flexibility, it might lose the very efficiency gains that justified compression in the first place.

The local workforce conversations that inform our thinking reinforce this dilemma rather than resolve it. In Tennessee, recent academic-practice discussions have emphasized student readiness in areas such as communication, policy, data literacy, professionalism, and applied translation of evidence (15). These priorities align with national employer data: Kedia et al. (4) analysis of 365 entry-level public health job postings found that oral and written communication skills were required in 92.3% of postings, health education and promotion skills in 85.2%, and cultural competency in 75.3%. Quantitative research methods, by contrast, appeared in fewer than 7% of postings. That pattern describes a workforce signal that is broad and integrative rather than technically narrow—a signal that a compressiondesign optimizing for a single function risks obscuring rather than amplifying. Those emphases support our view that undergraduate public health should remain practical and externally oriented. At the same time, they do not suggest that a shorter degree is automatically better aligned with practice. If anything, they suggest that meaningful workforce readiness depends on repeated exposure, scaffolding, and authentic application across the undergraduate experience. We do not present this context as representative of all BSPH programs; rather, we offer it as an example of how reduced-credit proposals quickly surface competing educational priorities that may remain less visible in abstract policy discussions.

## Discussion

The reduced-credit bachelor’s pathway introduced by SACSCOC deserves serious attention. It is a legitimate invitation to innovate, and some undergraduate public health programs may eventually design thoughtful 90- to 100-credit credentials that clearly target direct workforce entry and communicate their purpose transparently (8, 9). We do not dismiss that possibility. We do, however, caution against assuming that because the BSPH is applied, it is therefore easily compressible. The degree to which a reduced-credit BSPH is viable—and for whom—will vary substantially by institutional context. A research university BSPH with a co-located MPH program, a well-resourced advising infrastructure, and a student population that arrives with clear post-baccalaureate plans faces very different tradeoffs than a regional comprehensive university whose BSPH serves a mixed population of direct-entry students, community college transfers, career-changers, and students who discover public health mid-college. An HBCU or minority-serving institution BSPH may be simultaneously managing first-generation student support, economic barriers to extended enrollment, and a workforce development mission that is central rather than incidental to its institutional identity. These differences are not incidental. They are the reason generic prescriptions about whether the BSPH “can” be compressed are insufficient. The answer is almost certainly yes for some programs in some contexts, and almost certainly no for others—and the field needs a vocabulary for that distinction that it does not currently have.

The BSPH occupies an unstable but valuable position between workforce preparation and broader undergraduate formation. Its public health identity depends not only on specific content domains, but also on a curricular ecology that allows students to connect science, policy, data, communication, ethics, and social context. In many institutional settings, including our own, the degree also serves students whose professional intentions are not settled at the point of entry. That adaptability is part of its value. A shorter model may serve students who arrive with clear professional intentions, but it diminishes the degree for those who do not. That concern becomes especially important when reduced-credit or accelerated models are linked to graduate progression, because the student-friendliness of such designs depends not only on efficient advancement for successful students, but also on the quality of the off-ramp for those who do not continue as planned.

Our central argument, stated plainly, is this: a reduced-credit BSPH cannot simultaneously optimize for all of the functions the degree has traditionally served without accepting meaningful loss in at least one of them. For that reason, the central question should not be whether the BSPH can be made shorter. The more important question is what undergraduate public health should optimize for. If the answer is direct workforce entry, then programs should say so clearly and design accordingly. If the answer is broad preparation for multiple next steps, then compression may prove counterproductive. If the answer varies by institutional mission and student population, then public health educators should resist generic solutions and instead define reduced-credit proposals in relation to local realities, student pathways, and the distinct educational purposes of the degree (Table 1).

**Table 1:** A typology of BSPH model types and their implications for curriculum compression.

BSPH model type	Primary optimization target	CEPH curriculum domains emphasized	CEPH domains most vulnerable to compression
Workforce-Entry BSPH	Direct employment in community and public health practice settings at the bachelor’s level	Domains 9–10 (statistical literacy; data collection/surveillance); 11–12 (evidence-based intervention planning; partnerships); 13 (critical thinking); B4 workforce preparation concepts (professionalism, networking, leadership, cultural humility); B5 experiential learning as the primary workforce readiness signal	Domains 1 (PH history); 4 (ethics depth); 5–6 (biological science; disease foundations); 7–8 (health systems comparison; global health) — theoretical and scientific breadth most at risk when credits are reduced
Pre-Professional/Pre-Graduate BSPH	Preparation for graduate or professional programs (MPH, medicine, law, health policy)	Domains 3 (determinants); 5–6 (biological and disease science); 9–10 (data and quantitative methods); 13 (critical thinking); 15 (policy analysis); B2 foundational competencies (information evaluation and synthesis)	Domains 11–12 (applied intervention planning; community partnerships); B4 workforce preparation concepts; B5 experiential learning depth; breadth often sacrificed for depth in areas valued by graduate admissions processes
Liberal Arts/Population Health Hybrid BSPH	Broad undergraduate formation across the full scope of public health knowledge; adaptable to evolving student goals and multiple post-baccalaureate pathways	All 15 B1 domains treated as co-equal; particular emphasis on Domains 1–4 (history, social justice/equity, determinants, ethics) and 8 (global health) as connective tissue; B5 experiential learning serves integrative rather than purely vocational purposes	Breadth IS the value proposition: no domain hierarchy exists to exploit for compression without undermining the model’s core purpose. Any credit reduction directly compresses the adaptive capacity that defines the degree’s utility for its primary student population

Domain numbers refer to CEPH (2024) Criterion B1 required curriculum domains for standalone baccalaureate programs.

Several limitations of this perspective warrant acknowledgment. This paper is grounded primarily in our experience within a single CEPH-accredited BSPH program at a regional public university in Tennessee, and institutional context almost certainly shapes how we read the tradeoffs involved. Programs at research universities, urban institutions, historically Black colleges and universities, or community-college-transfer-serving institutions may weigh these considerations quite differently, a point we return to in the typology above. More consequentially, the empirical literature directly comparing student outcomes across full-credit and reduced-credit undergraduate public health programs does not yet exist. We do not argue from outcome evidence because no such evidence is available to argue from. We regard that absence itself as significant: the field is being invited to reform a degree structure before it can assess what is being lost or preserved. That is not an argument against innovation, but it is a strong argument for the kind of explicit purpose-setting we describe here.

In our view, the BSPH is a particularly complex case for reduced-credit design because its strengths often derive from its capacity to accommodate students whose goals and intentions develop across the degree. Innovation in undergraduate public health may indeed require new credential structures, but those structures should follow a clear statement of purpose rather than precede it. Before the field embraces reduced-credit BSPH models, programs should be prepared to state, with unusual candor, what they are preserving, what they are giving up, whether the credential is intended to stand alone or function as a graduate pipeline, and which students those tradeoffs are intended to serve. Our position is this: the BSPH should not be compressed without first answering—with unusual specificity—what the degree is designed to optimize for, and which students that choice is intended to serve.

## Data Availability

The original contributions presented in the study are included in the article/supplementary material, further inquiries can be directed to the corresponding author.
